# *rac*-[2-(Dicyclohexylphosphanyl)phenyl](phenyl)phosphinic diisopropyl­amide–borane hemihydrate

**DOI:** 10.1107/S1600536813001839

**Published:** 2013-01-23

**Authors:** Stephen J. Evans, C. Alicia Renison, D. Bradley G. Williams, Alfred Muller

**Affiliations:** a Research Centre for Synthesis and Catalysis, Department of Chemistry, University of Johannesburg (APK Campus), PO Box 524, Auckland Park, Johannesburg, 2006, South Africa

## Abstract

In the title compound, C_30_H_48_BNOP_2_·0.5H_2_O, the water molecule is disordered about an inversion centre. Both phospho­rus atoms shows distortions in their tetra­hedral environments with the cyclo­hexyl substituents disordered over two orientations in a 0.851 (3):0.149 (3) occupancy ratio. The crystal structure is assembled *via* O—H⋯O inter­actions between pairs of phosphininc amide mol­ecules and water molecules, creating hydrogen-bonded dimers with graph-set *R*_2_^4^(8) along [001]. Weak C—H⋯O inter­actions are also observed.

## Related literature

For background to the synthesis of ligands derived from phosphinic amides, see: Williams *et al.* (2009 ▶). For background to DoM technology, see: Snieckus (1990 ▶). For details of cone angles, see: Tolman (1977 ▶); Otto (2001 ▶). For graph-set notation, see: Bernstein *et al.* (1995 ▶).
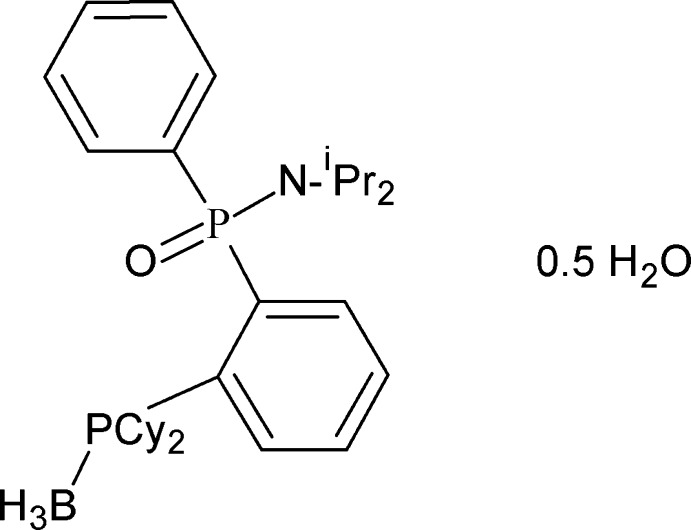


## Experimental

### 

#### Crystal data

C_30_H_48_BNOP_2_·0.5H_2_O
*M* *_r_* = 1040.9
Triclinic, 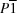

*a* = 11.2480 (3) Å
*b* = 11.5240 (3) Å
*c* = 14.1640 (4) Å
α = 90.543 (2)°
β = 108.178 (1)°
γ = 118.826 (1)°
*V* = 1499.73 (7) Å^3^
*Z* = 1
Mo *K*α radiation
μ = 0.17 mm^−1^
*T* = 100 K
0.25 × 0.17 × 0.12 mm

#### Data collection

Bruker X8 APEXII 4K KappaCCD diffractometer
34539 measured reflections
7448 independent reflections
5330 reflections with *I* > 2σ(*I*)
*R*_int_ = 0.053

#### Refinement

*R*[*F* ^2^ > 2σ(*F* ^2^)] = 0.050
*wR*(*F* ^2^) = 0.134
*S* = 1.04
7448 reflections
447 parameters
314 restraints
H atoms treated by a mixture of independent and constrained refinement
Δρ_max_ = 0.55 e Å^−3^
Δρ_min_ = −0.27 e Å^−3^

### 

Data collection: *APEX2* (Bruker, 2005 ▶); cell refinement: *SAINT-Plus* (Bruker, 2004 ▶); data reduction: *SAINT-Plus* and *XPREP* (Bruker, 2004 ▶); program(s) used to solve structure: *SIR97* (Altomare *et al.*, 1999 ▶); program(s) used to refine structure: *SHELXL97* (Sheldrick, 2008 ▶); molecular graphics: *DIAMOND* (Brandenburg & Putz, 2005 ▶); software used to prepare material for publication: *publCIF* (Westrip, 2010 ▶) and *WinGX* (Farrugia, 2012 ▶).

## Supplementary Material

Crystal structure: contains datablock(s) global, I. DOI: 10.1107/S1600536813001839/zq2191sup1.cif

Structure factors: contains datablock(s) I. DOI: 10.1107/S1600536813001839/zq2191Isup2.hkl

Additional supplementary materials:  crystallographic information; 3D view; checkCIF report

## Figures and Tables

**Table 1:** Hydrogen-bond geometry (Å, °)

*D*—H⋯*A*	*D*—H	H⋯*A*	*D*⋯*A*	*D*—H⋯*A*
O2—H7*B*⋯O1^i^	0.88 (7)	1.85 (7)	2.722 (4)	167 (6)
O2—H7*A*⋯O1	0.85 (5)	1.95 (5)	2.768 (4)	163 (5)
C51*A*—H51*A*⋯O1	1.00	2.28	3.083 (3)	136
C61*A*—H61*A*⋯O1	1.00	2.31	3.057 (5)	130

Symmetry code: (i) 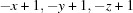.

**Fig. 1.**
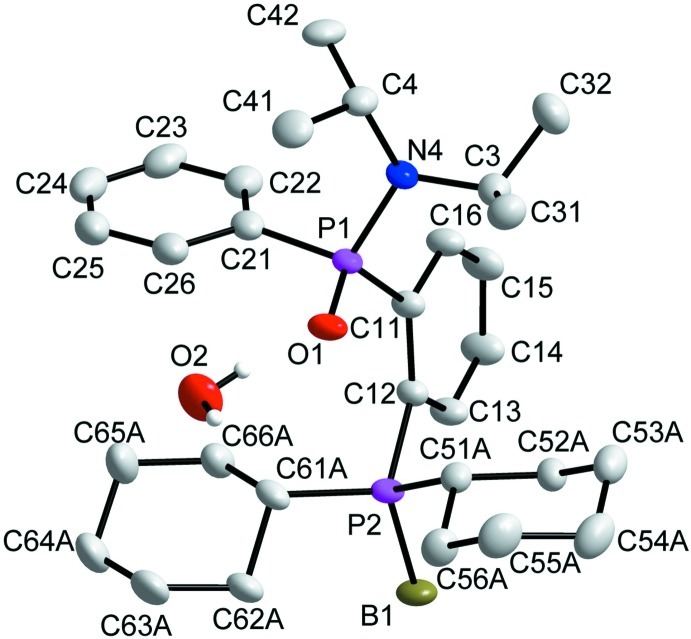
A view of the title complex, showing the atom-numbering scheme and 50% probability displacement ellipsoids. Hydrogen atoms (except for the water solvate) as well as the minor part of the disorder omitted for clarity.

**Fig. 2.**
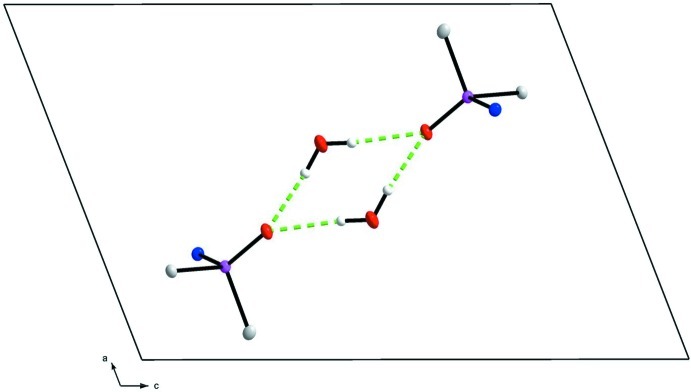
Packing diagram showing the O—H···O hydrogen bonding interactions (indicated by green dashed lines).

## supplementary crystallographic information

### Comment

An expedient rapid synthesis of ligands derived from phosphinic amides that were found to be suitable for the Suzuki-Miyaura reactions at low palladium catalyst loadings was developed (Williams *et al.*, 2009). The brief practical synthesis affords arylphosphine ligands resistant to oxidation and hydrolysis while maintaining high catalyst activity. The synthesis rests strongly on DoM technology (Snieckus, 1990) making use of a directing group that is highly underrepresented in this type of chemistry. We envisioned that the use of phosphinic amides as directing groups, together with phosphinous chloride (Cy_2_PCl) electrophiles would allow the synthesis of sterically hindered phosphines that are stable to hydrolysis and oxidation. The *ortho*-deprotonation of phosphinic amides with sec-butyl-lithium and quenching with dicyclohexylphosphinous chloride (Cy_2_PCl) allowed isolation of the desired ligand in good yields (45–60% yield), which are stable to air, liquid-liquid extraction, and chromatography without special exclusion of oxygen.

The title compound (Fig. 1) crystallizes in the triclinic space group *P*1 (*Z* = 2) with the asymmetric unit containing half a molecule of water as it is disordered over an inversion centre. Both the phosphorus centres show varying degrees of distortion in their tetrahedral environments, in particular towards the more bulky substituents, *i.e.* towards the amide for P1 [O1—P1—N4 = 117.44 (10)°] and (to lesser extend) towards one of the cyclohexyls for P2 [B1—P2—C61 = 112.59 (12)°].

The most common method used for determining the steric behaviour of a phosphane ligand is the Tolman cone angle (Tolman, 1977). We used the geometry from the title compound and adjusted the P═O and P—B distances to 2.28 Å (the average Ni—P distance used in the original Tolman model) to cancel the bias this may have on the calculated cone angle value. In this way we obtain effective cone angle (Otto, 2001) values of 231 and 181° for P1 and P2 respectively.

The structure is stabilized by strong intermolecular O—H···O hydrogen bonds formed between the phosphinic oxygen atom and the oxygen atom of the water molecule, creating head-to-head dimeric structures with the phosphinic amide molecules (Fig. 2) The graph set notation for this interaction is *R*_2_^4^(8) (Bernstein *et al.*, 1995) Additional weak C—H···O interactions are also observed and summarized in Table 1.

### Experimental

Cyclohexylchloride (1 mL, 8.42 mmol) was added to a solution of diethyl ether (10 ml) and magnesium turnings (1.0 eq., 204 mg, 8.42 mmol) along with one crystal of iodine as an initiator and the mixture was heated under reflux until all the magnesium had been consumed. In a separate flask, PCl_3_ (3.24 mmol, 0.38 eq., 283 µL) was dissolved in diethyl ether (40 mL) and the solution cooled to -40 °C. The cyclohexylmagnesium chloride solution was added dropwise over 10 minutes and the solution was allowed to warm to room temperature over three hours. Once the reaction was complete the salts that formed were filtered through a pad of Celite. The resultant product was approximately 70% pure (as determined by ^31^P NMR spectroscopy) and was used without further manipulation, the reaction producing (2.27 mmol) of chloro-dicyclohexylphosphine.

*N,N*-Diisopropyldiphenylphosphinic amide (569 mg, 1.89 mmol) was weighed out in a Schlenk flask and THF (10 mL) was added. The solution was then cooled to -60 °C and sec-BuLi (1.1 eq., 1*M*) was added. The solution was allowed to stir for three hours between -40 and -70 °C after which it was cooled to -78 °C and the electrophile (1.2 eq.) dissolved in a small amount of THF was added. The reaction mixture was allowed to warm to room temperature over four hours and was stirred at room temperature overnight. All solvents were then removed *in vacuo* and the residue was extracted with EtOAc and H_2_O. The product was purified by column chromatography on flash silica.

Protection of the phosphine occurred by first dissolving the phosphine in THF (10 mL) cooling the mixture to 0 °C and adding an excess of BH_3_ in THF and the reaction stirred at room temperature for 5 h. All solvents were then removed *in vacuo* and the resulted residue was the desired product in 100% yield. Crystals were grown by dissolving the ligand in a minimal amount of DCM and then layering an excess of hexane on the DCM and allowing to stand in a refrigerator until the crystals were formed.

Yield: 51% (White solid).

^1^H NMR: (300 MHz, CDCl_3_) δH 7.94 — 7.87 (m, 1H, H3), 7.69 — 7.61 (m, 1H, H6), 7.60 (dd, 2H, H2` and H6`, *J* = 11.7 and 7.5 Hz), 7.50 — 7.33 (m, 5H, aromatic), 3.49 and 3.43 (2×sept, 2H, NCH(CH_3_)_2_, *J* = 6.6 Hz), 2.03 — 1.22 (m, 22H, aliphatic), 1.37 and 1.15 (2×d, 12H, NCH(CH_3_)_2_, *J* = 6.6 Hz). ^13^C NMR: (75 MHz, CDCl_3_) δC 140.0 (dd, 1 C, C2, *J* = 31.3 and 14.0 Hz), 140.8 (dd, 1 C, C1, *J* = 124.9 and 28.8 Hz), 137.2 (dd, 1 C, C1`, *J* = 121.8 and 1.1 Hz), 133.6 (d, 1 C, C3, *J* = 12.4 Hz), 132.6 (dd. 1 C, C6, *J* = 11.5 and 8.1 Hz), 131.5 (d, 2 C, C3` and C5`, *J* = 9.8 Hz), 130.0 (d, 1 C, C4, *J* = 2.6 Hz), 129.6 (d. 1 C, C4`, *J* = 2.6 Hz), 127.3 (d, 2 C, C2` and C6`, *J* = 12.7 Hz), 126.9 (d. 1 C, C5, *J* = 12.1 Hz), 46.8 (d, 2 C, NCH_2_(CH_3_)_2_, *J* = 4.6 Hz), 35.5 (dd, 1 C, C1``, *J* = 109.1 and 18.4 Hz), 30.3–23.3 (m, 1 C, aliphatic), 23.0 (d, 4 C, NCH(CH_3_)_2_, *J* = 2.0 Hz). ^31^P NMR: (121 MHz, CDCl_3_) δP 33.5 (d, 1P, P(O)N, *J* = 10.5 Hz), 5.0 (Br s, 1P, BH_3_—PCy_2_). IR: (CHCl_3_/cm^-1^) 3015, 2402, 1524, 722 CIMS: *m/z* 497 [(M—BH_2_), 10%], 414 [(M—C_6_H_11_—BH_3_), 100%].

### Refinement

The aromatic, methine, methylene, methyl and BH_3_ hydrogen atoms were placed in geometrically idealized positions (C—H = 0.95–1.0 Å, B—H = 0.98 Å) O—H = 0.87 Å) and constrained to ride on their parent atoms with *U*_iso_(H) = 1.2*U*_eq_(C) for the aromatic, methine and methylene H and *U*_iso_(H) = 1.5*U*_eq_(C) for the methyl and B—H respectively. Locations of the methyl hydrogen atoms were initially obtained from a Fourier difference map and refined as a fixed rotor. Refinement of the oxygen atom of the water molecule showed large thermal vibration, and in subsequent refinement cycles the occupancy thereof was freed. This refined to nearly 50% and in the final refinement cycles the occupancy value was constrained to half. The hydrogen atoms of the water molecule were located from a Fourier difference map. Both of the cyclohexcyl substituents showed somewhat large thermal ellipsoids and were subsequently refined as disordered over two positions. Their geometries and ellipsoid sizes were kept reasonable by restraining with the appropriate refinement commands (SAME, SADI and SIMU). The occupancies were refined with a free variable that added to unity and a final ratio of 85:15 was obtained between the two components. Discrepant reflection 001 was removed in the final stages of refinement.

### Crystal data

**Table:**

C_30_H_48_BNOP_2_·0.5H_2_O	*Z* = 1
*M**_r_* = 1040.9	*F*(000) = 566
Triclinic, *P*1	*D*_x_ = 1.153 Mg m^−^^3^
Hall symbol: -P 1	Mo *K*α radiation, λ = 0.71069 Å
*a* = 11.2480 (3) Å	Cell parameters from 5800 reflections
*b* = 11.5240 (3) Å	θ = 2.2–25.8°
*c* = 14.1640 (4) Å	µ = 0.17 mm^−^^1^
α = 90.543 (2)°	*T* = 100 K
β = 108.178 (1)°	Prism, colourless
γ = 118.826 (1)°	0.25 × 0.17 × 0.12 mm
*V* = 1499.73 (7) Å^3^	

### Data collection

**Table:**

Bruker X8 APEXII 4K KappaCCD diffractometer	5330 reflections with *I* > 2σ(*I*)
Graphite monochromator	*R*_int_ = 0.053
Detector resolution: 8.4 pixels mm^-1^	θ_max_ = 28.3°, θ_min_ = 2.1°
φ and ω scans	*h* = −14→15
34539 measured reflections	*k* = −15→15
7448 independent reflections	*l* = −18→18

### Refinement

**Table:**

Refinement on *F*^2^	Primary atom site location: structure-invariant direct methods
Least-squares matrix: full	Secondary atom site location: difference Fourier map
*R*[*F*^2^ > 2σ(*F*^2^)] = 0.050	Hydrogen site location: inferred from neighbouring sites
*wR*(*F*^2^) = 0.134	H atoms treated by a mixture of independent and constrained refinement
*S* = 1.04	*w* = 1/[σ^2^(*F*_o_^2^) + (0.058*P*)^2^ + 0.5817*P*] where *P* = (*F*_o_^2^ + 2*F*_c_^2^)/3
7448 reflections	(Δ/σ)_max_ = 0.001
447 parameters	Δρ_max_ = 0.55 e Å^−^^3^
314 restraints	Δρ_min_ = −0.27 e Å^−^^3^

### Special details

**Table:**

Experimental. The intensity data was collected on a Bruker X8 APEXII 4 K KappaCCD diffractometer using an exposure time of 20 s/frame. A total of 2529 frames were collected with a frame width of 0.5° covering up to θ = 28.33° with 99.6% completeness accomplished.
Geometry. All e.s.d.'s (except the e.s.d. in the dihedral angle between two l.s. planes) are estimated using the full covariance matrix. The cell e.s.d.'s are taken into account individually in the estimation of e.s.d.'s in distances, angles and torsion angles; correlations between e.s.d.'s in cell parameters are only used when they are defined by crystal symmetry. An approximate (isotropic) treatment of cell e.s.d.'s is used for estimating e.s.d.'s involving l.s. planes.
Refinement. Refinement of *F*^2^ against ALL reflections. The weighted *R*-factor *wR* and goodness of fit *S* are based on *F*^2^, conventional *R*-factors *R* are based on *F*, with *F* set to zero for negative *F*^2^. The threshold expression of *F*^2^ > σ(*F*^2^) is used only for calculating *R*-factors(gt) *etc*. and is not relevant to the choice of reflections for refinement. *R*-factors based on *F*^2^ are statistically about twice as large as those based on *F*, and *R*- factors based on ALL data will be even larger.

### Fractional atomic coordinates and isotropic or equivalent isotropic displacement parameters (Å^2^)

**Table:**

	*x*	*y*	*z*	*U*_iso_*/*U*_eq_	Occ. (<1)
P1	0.73833 (5)	0.64726 (5)	0.78185 (4)	0.02098 (13)	
P2	0.60597 (5)	0.28217 (5)	0.75649 (4)	0.02207 (13)	
O1	0.63962 (15)	0.56005 (13)	0.68112 (10)	0.0288 (3)	
N4	0.70251 (17)	0.75742 (16)	0.82270 (12)	0.0257 (4)	
C3	0.5686 (2)	0.6994 (2)	0.84861 (16)	0.0308 (5)	
H3	0.5525	0.6122	0.8701	0.037*	
C4	0.7405 (2)	0.8879 (2)	0.78540 (16)	0.0304 (5)	
H4	0.6723	0.9148	0.7952	0.036*	
C11	0.7507 (2)	0.55277 (19)	0.88326 (14)	0.0241 (4)	
C12	0.71675 (19)	0.41644 (18)	0.87099 (14)	0.0230 (4)	
C13	0.7584 (2)	0.3688 (2)	0.95869 (15)	0.0288 (4)	
H13	0.739	0.2786	0.9519	0.035*	
C14	0.8263 (2)	0.4469 (2)	1.05460 (16)	0.0351 (5)	
H14	0.8554	0.4114	1.1119	0.042*	
C15	0.8515 (2)	0.5762 (2)	1.06664 (16)	0.0345 (5)	
H15	0.8937	0.6294	1.1324	0.041*	
C16	0.8147 (2)	0.6280 (2)	0.98160 (15)	0.0298 (4)	
H16	0.8335	0.7179	0.9904	0.036*	
C21	0.9236 (2)	0.7385 (2)	0.78428 (16)	0.0291 (4)	
C22	1.0458 (2)	0.8007 (2)	0.87218 (18)	0.0355 (5)	
H22	1.0346	0.8015	0.9359	0.043*	
C23	1.1844 (2)	0.8617 (2)	0.8683 (2)	0.0426 (6)	
H23	1.2675	0.9058	0.929	0.051*	
C24	1.2010 (3)	0.8581 (2)	0.7754 (2)	0.0495 (7)	
H24	1.2954	0.8977	0.7722	0.059*	
C25	1.0792 (3)	0.7966 (2)	0.6878 (2)	0.0463 (6)	
H25	1.0907	0.7961	0.6241	0.056*	
C26	0.9422 (3)	0.7363 (2)	0.69121 (19)	0.0385 (5)	
H26	0.8596	0.6928	0.6303	0.046*	
C31	0.4312 (2)	0.6669 (2)	0.75826 (17)	0.0359 (5)	
H31A	0.4402	0.7509	0.7379	0.054*	
H31B	0.3458	0.6204	0.7779	0.054*	
H31C	0.4201	0.6086	0.7014	0.054*	
C32	0.5886 (3)	0.7900 (2)	0.93776 (17)	0.0403 (5)	
H32A	0.6768	0.8101	0.9944	0.06*	
H32B	0.5041	0.744	0.9585	0.06*	
H32C	0.5977	0.8743	0.9179	0.06*	
C41	0.7158 (3)	0.8768 (2)	0.67253 (17)	0.0390 (5)	
H41A	0.7899	0.8638	0.6601	0.059*	
H41B	0.7227	0.9598	0.6512	0.059*	
H41C	0.619	0.7998	0.6339	0.059*	
C42	0.8933 (2)	1.0017 (2)	0.84945 (19)	0.0377 (5)	
H42A	0.9068	1.0037	0.9214	0.057*	
H42B	0.9058	1.0881	0.8323	0.057*	
H42C	0.9653	0.9865	0.8358	0.057*	
C51A	0.4322 (2)	0.2804 (3)	0.7140 (2)	0.0250 (6)	0.851 (3)
H51A	0.4547	0.3739	0.7053	0.03*	0.851 (3)
C52A	0.3642 (3)	0.2451 (3)	0.7958 (2)	0.0300 (6)	0.851 (3)
H52A	0.3482	0.1558	0.8102	0.036*	0.851 (3)
H52B	0.4322	0.3129	0.859	0.036*	0.851 (3)
C53A	0.2193 (3)	0.2417 (3)	0.7622 (3)	0.0391 (6)	0.851 (3)
H53A	0.1755	0.2139	0.8147	0.047*	0.851 (3)
H53B	0.2367	0.3332	0.7545	0.047*	0.851 (3)
C54A	0.1149 (3)	0.1443 (3)	0.6627 (3)	0.0452 (8)	0.851 (3)
H54A	0.0227	0.145	0.6418	0.054*	0.851 (3)
H54B	0.0928	0.0517	0.6712	0.054*	0.851 (3)
C55A	0.1812 (3)	0.1839 (3)	0.5812 (2)	0.0443 (7)	0.851 (3)
H55A	0.1982	0.2745	0.5701	0.053*	0.851 (3)
H55B	0.1122	0.1189	0.5168	0.053*	0.851 (3)
C56A	0.3250 (3)	0.1857 (3)	0.6117 (2)	0.0343 (6)	0.851 (3)
H56A	0.3683	0.216	0.5592	0.041*	0.851 (3)
H56B	0.3066	0.0932	0.6164	0.041*	0.851 (3)
C51B	0.4299 (11)	0.2635 (18)	0.6799 (11)	0.033 (2)	0.149 (3)
H51B	0.4438	0.3364	0.6393	0.039*	0.149 (3)
C52B	0.3637 (15)	0.2733 (17)	0.7555 (13)	0.0363 (19)	0.149 (3)
H52C	0.4275	0.3627	0.8011	0.044*	0.149 (3)
H52D	0.3552	0.2032	0.7972	0.044*	0.149 (3)
C53B	0.2107 (14)	0.2536 (16)	0.6988 (14)	0.042 (2)	0.149 (3)
H53C	0.1644	0.2496	0.7487	0.05*	0.149 (3)
H53D	0.2227	0.3333	0.6677	0.05*	0.149 (3)
C54B	0.1124 (17)	0.131 (2)	0.6188 (14)	0.041 (2)	0.149 (3)
H54C	0.027	0.1357	0.5767	0.049*	0.149 (3)
H54D	0.077	0.0509	0.651	0.049*	0.149 (3)
C55B	0.1864 (14)	0.1125 (18)	0.5510 (11)	0.042 (2)	0.149 (3)
H55C	0.1206	0.0229	0.5057	0.051*	0.149 (3)
H55D	0.2032	0.1819	0.5081	0.051*	0.149 (3)
C56B	0.3309 (14)	0.1234 (16)	0.6109 (12)	0.035 (2)	0.149 (3)
H56C	0.3762	0.1125	0.5643	0.042*	0.149 (3)
H56D	0.3156	0.0521	0.6521	0.042*	0.149 (3)
C61A	0.6890 (3)	0.3241 (4)	0.65939 (19)	0.0264 (6)	0.851 (3)
H61A	0.664	0.3876	0.6226	0.032*	0.851 (3)
C62A	0.6305 (3)	0.1964 (3)	0.5823 (2)	0.0338 (6)	0.851 (3)
H62A	0.6542	0.1321	0.6174	0.041*	0.851 (3)
H62B	0.5234	0.1517	0.5498	0.041*	0.851 (3)
C63A	0.6999 (3)	0.2363 (3)	0.5022 (2)	0.0452 (7)	0.851 (3)
H63A	0.663	0.1545	0.4528	0.054*	0.851 (3)
H63B	0.6719	0.297	0.4653	0.054*	0.851 (3)
C64A	0.8652 (4)	0.3072 (4)	0.5497 (3)	0.0506 (8)	0.851 (3)
H64A	0.8939	0.2437	0.5809	0.061*	0.851 (3)
H64B	0.9075	0.3363	0.4966	0.061*	0.851 (3)
C65A	0.9237 (4)	0.4289 (3)	0.6295 (3)	0.0503 (8)	0.851 (3)
H65A	0.9062	0.4973	0.5963	0.06*	0.851 (3)
H65B	1.0301	0.4692	0.6632	0.06*	0.851 (3)
C66A	0.8532 (3)	0.3931 (3)	0.7089 (2)	0.0357 (7)	0.851 (3)
H66A	0.8793	0.3325	0.7476	0.043*	0.851 (3)
H66B	0.8902	0.4763	0.757	0.043*	0.851 (3)
C61B	0.7166 (16)	0.335 (3)	0.6756 (11)	0.030 (2)	0.149 (3)
H61B	0.7345	0.4276	0.6682	0.037*	0.149 (3)
C62B	0.6575 (16)	0.2600 (18)	0.5653 (11)	0.0366 (19)	0.149 (3)
H62C	0.632	0.1651	0.5667	0.044*	0.149 (3)
H62D	0.5666	0.2591	0.5275	0.044*	0.149 (3)
C63B	0.7597 (17)	0.318 (2)	0.5077 (11)	0.044 (2)	0.149 (3)
H63C	0.7678	0.4049	0.4923	0.053*	0.149 (3)
H63D	0.717	0.2559	0.4424	0.053*	0.149 (3)
C64B	0.9113 (18)	0.343 (2)	0.5639 (14)	0.045 (2)	0.149 (3)
H64C	0.9067	0.2551	0.5667	0.054*	0.149 (3)
H64D	0.9754	0.3933	0.5264	0.054*	0.149 (3)
C65B	0.9745 (17)	0.4207 (18)	0.6688 (13)	0.041 (2)	0.149 (3)
H65C	0.9963	0.5145	0.6662	0.049*	0.149 (3)
H65D	1.0669	0.424	0.7061	0.049*	0.149 (3)
C66B	0.8695 (18)	0.3561 (19)	0.7245 (13)	0.033 (2)	0.149 (3)
H66C	0.9147	0.4127	0.7928	0.039*	0.149 (3)
H66D	0.8592	0.2671	0.7338	0.039*	0.149 (3)
B1	0.5842 (3)	0.1134 (2)	0.79117 (19)	0.0307 (5)	
H1A	0.5426	0.0925	0.8444	0.046*	
H1B	0.6796	0.1205	0.8156	0.046*	
H1C	0.5193	0.0412	0.7312	0.046*	
O2	0.6069 (4)	0.5108 (4)	0.4803 (3)	0.0472 (9)	0.5
H7A	0.607 (5)	0.535 (5)	0.537 (4)	0.036 (14)*	0.5
H7B	0.520 (7)	0.477 (6)	0.432 (5)	0.068 (19)*	0.5

### Atomic displacement parameters (Å^2^)

**Table:**

	*U*^11^	*U*^22^	*U*^33^	*U*^12^	*U*^13^	*U*^23^
P1	0.0277 (2)	0.0175 (2)	0.0217 (3)	0.01398 (19)	0.00962 (19)	0.00457 (18)
P2	0.0256 (2)	0.0174 (2)	0.0231 (3)	0.01272 (19)	0.00571 (19)	0.00282 (18)
O1	0.0438 (8)	0.0212 (7)	0.0208 (7)	0.0187 (6)	0.0076 (6)	0.0038 (5)
N4	0.0326 (8)	0.0230 (8)	0.0288 (9)	0.0182 (7)	0.0130 (7)	0.0063 (7)
C3	0.0360 (10)	0.0287 (11)	0.0372 (12)	0.0202 (9)	0.0184 (9)	0.0117 (9)
C4	0.0336 (10)	0.0234 (10)	0.0405 (12)	0.0183 (8)	0.0148 (9)	0.0090 (9)
C11	0.0305 (9)	0.0214 (9)	0.0215 (10)	0.0151 (8)	0.0077 (7)	0.0041 (7)
C12	0.0275 (9)	0.0210 (9)	0.0229 (10)	0.0143 (7)	0.0086 (7)	0.0037 (7)
C13	0.0414 (11)	0.0223 (10)	0.0265 (11)	0.0199 (9)	0.0105 (9)	0.0064 (8)
C14	0.0529 (13)	0.0313 (11)	0.0234 (11)	0.0263 (10)	0.0083 (9)	0.0083 (9)
C15	0.0473 (12)	0.0341 (12)	0.0222 (11)	0.0242 (10)	0.0070 (9)	0.0029 (9)
C16	0.0396 (11)	0.0228 (10)	0.0270 (11)	0.0177 (9)	0.0090 (9)	0.0035 (8)
C21	0.0393 (11)	0.0240 (10)	0.0347 (11)	0.0209 (9)	0.0184 (9)	0.0110 (8)
C22	0.0380 (11)	0.0279 (11)	0.0481 (14)	0.0210 (9)	0.0176 (10)	0.0107 (10)
C23	0.0365 (11)	0.0267 (11)	0.0637 (17)	0.0161 (9)	0.0168 (11)	0.0135 (11)
C24	0.0471 (14)	0.0302 (12)	0.088 (2)	0.0218 (11)	0.0412 (14)	0.0166 (13)
C25	0.0608 (15)	0.0315 (12)	0.0611 (17)	0.0233 (11)	0.0400 (14)	0.0107 (12)
C26	0.0511 (13)	0.0276 (11)	0.0461 (14)	0.0215 (10)	0.0267 (11)	0.0099 (10)
C31	0.0355 (11)	0.0321 (11)	0.0387 (13)	0.0170 (9)	0.0120 (9)	0.0087 (9)
C32	0.0497 (13)	0.0486 (14)	0.0362 (13)	0.0320 (11)	0.0203 (10)	0.0098 (11)
C41	0.0516 (13)	0.0363 (12)	0.0416 (13)	0.0298 (11)	0.0193 (11)	0.0201 (10)
C42	0.0372 (11)	0.0193 (10)	0.0559 (15)	0.0154 (9)	0.0141 (10)	0.0043 (10)
C51A	0.0274 (10)	0.0239 (11)	0.0286 (13)	0.0158 (8)	0.0113 (9)	0.0094 (11)
C52A	0.0350 (11)	0.0291 (12)	0.0366 (14)	0.0199 (9)	0.0201 (10)	0.0118 (10)
C53A	0.0389 (12)	0.0375 (13)	0.0571 (17)	0.0247 (10)	0.0282 (12)	0.0198 (12)
C54A	0.0298 (11)	0.0462 (15)	0.0613 (18)	0.0201 (10)	0.0173 (13)	0.0224 (15)
C55A	0.0287 (11)	0.0478 (15)	0.0492 (16)	0.0189 (11)	0.0061 (11)	0.0128 (13)
C56A	0.0274 (10)	0.0370 (14)	0.0338 (12)	0.0153 (10)	0.0073 (9)	0.0064 (11)
C51B	0.029 (3)	0.031 (3)	0.035 (3)	0.015 (2)	0.009 (3)	0.010 (3)
C52B	0.035 (2)	0.032 (2)	0.044 (3)	0.018 (2)	0.015 (2)	0.010 (2)
C53B	0.032 (2)	0.042 (3)	0.057 (3)	0.025 (2)	0.016 (3)	0.012 (3)
C54B	0.029 (3)	0.044 (3)	0.052 (3)	0.021 (2)	0.013 (3)	0.012 (3)
C55B	0.030 (3)	0.043 (3)	0.045 (3)	0.015 (3)	0.008 (3)	0.010 (3)
C56B	0.027 (3)	0.035 (3)	0.038 (3)	0.015 (3)	0.007 (3)	0.008 (3)
C61A	0.0375 (13)	0.0288 (12)	0.0232 (12)	0.0243 (12)	0.0111 (10)	0.0051 (11)
C62A	0.0447 (13)	0.0281 (13)	0.0335 (13)	0.0209 (11)	0.0162 (10)	0.0003 (11)
C63A	0.0618 (16)	0.0385 (15)	0.0406 (14)	0.0247 (13)	0.0268 (12)	−0.0010 (12)
C64A	0.0579 (18)	0.0484 (18)	0.0600 (17)	0.0282 (14)	0.0376 (15)	0.0018 (14)
C65A	0.0438 (15)	0.0472 (15)	0.0622 (19)	0.0167 (12)	0.0332 (13)	−0.0007 (14)
C66A	0.0325 (12)	0.0349 (15)	0.0424 (15)	0.0172 (10)	0.0170 (11)	0.0013 (12)
C61B	0.040 (3)	0.030 (3)	0.032 (3)	0.025 (3)	0.014 (3)	0.004 (3)
C62B	0.046 (2)	0.034 (2)	0.035 (2)	0.024 (2)	0.016 (2)	0.004 (2)
C63B	0.052 (3)	0.042 (3)	0.044 (3)	0.022 (3)	0.027 (3)	0.003 (3)
C64B	0.046 (3)	0.042 (3)	0.053 (3)	0.021 (3)	0.027 (3)	0.005 (3)
C65B	0.039 (3)	0.038 (3)	0.046 (3)	0.017 (3)	0.022 (3)	0.005 (3)
C66B	0.035 (3)	0.031 (3)	0.037 (3)	0.019 (3)	0.016 (3)	0.005 (3)
B1	0.0381 (12)	0.0195 (11)	0.0349 (13)	0.0176 (9)	0.0089 (10)	0.0056 (9)
O2	0.053 (2)	0.063 (2)	0.0239 (18)	0.0325 (19)	0.0070 (16)	0.0043 (16)

### Geometric parameters (Å, º)

**Table:**

P1—O1	1.4788 (14)	C54A—H54B	0.99
P1—N4	1.6534 (16)	C55A—C56A	1.529 (4)
P1—C21	1.814 (2)	C55A—H55A	0.99
P1—C11	1.822 (2)	C55A—H55B	0.99
P2—C61A	1.837 (2)	C56A—H56A	0.99
P2—C51B	1.841 (5)	C56A—H56B	0.99
P2—C61B	1.845 (5)	C51B—C52B	1.512 (16)
P2—C51A	1.846 (2)	C51B—C56B	1.536 (16)
P2—C12	1.8465 (19)	C51B—H51B	1
P2—B1	1.929 (2)	C52B—C53B	1.554 (15)
N4—C3	1.493 (3)	C52B—H52C	0.99
N4—C4	1.503 (3)	C52B—H52D	0.99
C3—C32	1.519 (3)	C53B—C54B	1.479 (16)
C3—C31	1.538 (3)	C53B—H53C	0.99
C3—H3	1	C53B—H53D	0.99
C4—C42	1.526 (3)	C54B—C55B	1.524 (16)
C4—C41	1.528 (3)	C54B—H54C	0.99
C4—H4	1	C54B—H54D	0.99
C11—C16	1.400 (3)	C55B—C56B	1.523 (15)
C11—C12	1.421 (3)	C55B—H55C	0.99
C12—C13	1.401 (3)	C55B—H55D	0.99
C13—C14	1.380 (3)	C56B—H56C	0.99
C13—H13	0.95	C56B—H56D	0.99
C14—C15	1.374 (3)	C61A—C66A	1.516 (4)
C14—H14	0.95	C61A—C62A	1.542 (4)
C15—C16	1.386 (3)	C61A—H61A	1
C15—H15	0.95	C62A—C63A	1.525 (4)
C16—H16	0.95	C62A—H62A	0.99
C21—C22	1.385 (3)	C62A—H62B	0.99
C21—C26	1.400 (3)	C63A—C64A	1.523 (4)
C22—C23	1.386 (3)	C63A—H63A	0.99
C22—H22	0.95	C63A—H63B	0.99
C23—C24	1.389 (4)	C64A—C65A	1.514 (4)
C23—H23	0.95	C64A—H64A	0.99
C24—C25	1.380 (4)	C64A—H64B	0.99
C24—H24	0.95	C65A—C66A	1.522 (4)
C25—C26	1.369 (3)	C65A—H65A	0.99
C25—H25	0.95	C65A—H65B	0.99
C26—H26	0.95	C66A—H66A	0.99
C31—H31A	0.98	C66A—H66B	0.99
C31—H31B	0.98	C61B—C66B	1.532 (17)
C31—H31C	0.98	C61B—C62B	1.550 (15)
C32—H32A	0.98	C61B—H61B	1
C32—H32B	0.98	C62B—C63B	1.514 (15)
C32—H32C	0.98	C62B—H62C	0.99
C41—H41A	0.98	C62B—H62D	0.99
C41—H41B	0.98	C63B—C64B	1.518 (16)
C41—H41C	0.98	C63B—H63C	0.99
C42—H42A	0.98	C63B—H63D	0.99
C42—H42B	0.98	C64B—C65B	1.489 (16)
C42—H42C	0.98	C64B—H64C	0.99
C51A—C56A	1.534 (3)	C64B—H64D	0.99
C51A—C52A	1.534 (4)	C65B—C66B	1.522 (15)
C51A—H51A	1	C65B—H65C	0.99
C52A—C53A	1.529 (3)	C65B—H65D	0.99
C52A—H52A	0.99	C66B—H66C	0.99
C52A—H52B	0.99	C66B—H66D	0.99
C53A—C54A	1.513 (4)	B1—H1A	0.98
C53A—H53A	0.99	B1—H1B	0.98
C53A—H53B	0.99	B1—H1C	0.98
C54A—C55A	1.521 (4)	O2—H7A	0.85 (5)
C54A—H54A	0.99	O2—H7B	0.88 (7)
			
O1—P1—N4	117.42 (8)	H55A—C55A—H55B	108
O1—P1—C21	109.44 (9)	C55A—C56A—C51A	110.9 (2)
N4—P1—C21	108.16 (9)	C55A—C56A—H56A	109.5
O1—P1—C11	113.32 (8)	C51A—C56A—H56A	109.5
N4—P1—C11	104.61 (9)	C55A—C56A—H56B	109.5
C21—P1—C11	102.77 (9)	C51A—C56A—H56B	109.5
C61A—P2—C51B	97.4 (6)	H56A—C56A—H56B	108
C51B—P2—C61B	105.8 (9)	C52B—C51B—C56B	109.0 (13)
C61A—P2—C51A	110.78 (14)	C52B—C51B—P2	105.3 (9)
C61B—P2—C51A	118.6 (7)	C56B—C51B—P2	109.7 (9)
C61A—P2—C12	111.28 (12)	C52B—C51B—H51B	110.9
C51B—P2—C12	116.4 (5)	C56B—C51B—H51B	110.9
C61B—P2—C12	104.2 (7)	P2—C51B—H51B	110.9
C51A—P2—C12	102.55 (11)	C51B—C52B—C53B	109.9 (11)
C61A—P2—B1	109.57 (17)	C51B—C52B—H52C	109.7
C51B—P2—B1	111.7 (6)	C53B—C52B—H52C	109.7
C61B—P2—B1	108.3 (10)	C51B—C52B—H52D	109.7
C51A—P2—B1	112.63 (12)	C53B—C52B—H52D	109.7
C12—P2—B1	109.89 (10)	H52C—C52B—H52D	108.2
C3—N4—C4	114.18 (15)	C54B—C53B—C52B	114.1 (12)
C3—N4—P1	115.94 (13)	C54B—C53B—H53C	108.7
C4—N4—P1	121.98 (13)	C52B—C53B—H53C	108.7
N4—C3—C32	111.47 (17)	C54B—C53B—H53D	108.7
N4—C3—C31	113.27 (17)	C52B—C53B—H53D	108.7
C32—C3—C31	110.55 (18)	H53C—C53B—H53D	107.6
N4—C3—H3	107.1	C53B—C54B—C55B	112.7 (14)
C32—C3—H3	107.1	C53B—C54B—H54C	109.1
C31—C3—H3	107.1	C55B—C54B—H54C	109.1
N4—C4—C42	112.22 (17)	C53B—C54B—H54D	109.1
N4—C4—C41	114.47 (17)	C55B—C54B—H54D	109.1
C42—C4—C41	111.57 (19)	H54C—C54B—H54D	107.8
N4—C4—H4	105.9	C56B—C55B—C54B	112.7 (13)
C42—C4—H4	105.9	C56B—C55B—H55C	109.1
C41—C4—H4	105.9	C54B—C55B—H55C	109.1
C16—C11—C12	118.45 (18)	C56B—C55B—H55D	109.1
C16—C11—P1	115.44 (14)	C54B—C55B—H55D	109.1
C12—C11—P1	125.71 (14)	H55C—C55B—H55D	107.8
C13—C12—C11	117.30 (17)	C55B—C56B—C51B	107.5 (11)
C13—C12—P2	112.72 (14)	C55B—C56B—H56C	110.2
C11—C12—P2	129.60 (14)	C51B—C56B—H56C	110.2
C14—C13—C12	122.96 (19)	C55B—C56B—H56D	110.2
C14—C13—H13	118.5	C51B—C56B—H56D	110.2
C12—C13—H13	118.5	H56C—C56B—H56D	108.5
C15—C14—C13	119.58 (19)	C66A—C61A—C62A	110.0 (3)
C15—C14—H14	120.2	C66A—C61A—P2	109.94 (19)
C13—C14—H14	120.2	C62A—C61A—P2	111.0 (2)
C14—C15—C16	119.17 (19)	C66A—C61A—H61A	108.6
C14—C15—H15	120.4	C62A—C61A—H61A	108.6
C16—C15—H15	120.4	P2—C61A—H61A	108.6
C15—C16—C11	122.38 (19)	C63A—C62A—C61A	109.1 (2)
C15—C16—H16	118.8	C63A—C62A—H62A	109.9
C11—C16—H16	118.8	C61A—C62A—H62A	109.9
C22—C21—C26	118.8 (2)	C63A—C62A—H62B	109.9
C22—C21—P1	124.06 (17)	C61A—C62A—H62B	109.9
C26—C21—P1	116.97 (16)	H62A—C62A—H62B	108.3
C21—C22—C23	120.8 (2)	C64A—C63A—C62A	111.3 (3)
C21—C22—H22	119.6	C64A—C63A—H63A	109.4
C23—C22—H22	119.6	C62A—C63A—H63A	109.4
C22—C23—C24	119.7 (2)	C64A—C63A—H63B	109.4
C22—C23—H23	120.2	C62A—C63A—H63B	109.4
C24—C23—H23	120.2	H63A—C63A—H63B	108
C25—C24—C23	119.5 (2)	C65A—C64A—C63A	110.3 (3)
C25—C24—H24	120.2	C65A—C64A—H64A	109.6
C23—C24—H24	120.2	C63A—C64A—H64A	109.6
C26—C25—C24	121.0 (2)	C65A—C64A—H64B	109.6
C26—C25—H25	119.5	C63A—C64A—H64B	109.6
C24—C25—H25	119.5	H64A—C64A—H64B	108.1
C25—C26—C21	120.2 (2)	C64A—C65A—C66A	112.4 (3)
C25—C26—H26	119.9	C64A—C65A—H65A	109.1
C21—C26—H26	119.9	C66A—C65A—H65A	109.1
C3—C31—H31A	109.5	C64A—C65A—H65B	109.1
C3—C31—H31B	109.5	C66A—C65A—H65B	109.1
H31A—C31—H31B	109.5	H65A—C65A—H65B	107.9
C3—C31—H31C	109.5	C61A—C66A—C65A	110.6 (3)
H31A—C31—H31C	109.5	C61A—C66A—H66A	109.5
H31B—C31—H31C	109.5	C65A—C66A—H66A	109.5
C3—C32—H32A	109.5	C61A—C66A—H66B	109.5
C3—C32—H32B	109.5	C65A—C66A—H66B	109.5
H32A—C32—H32B	109.5	H66A—C66A—H66B	108.1
C3—C32—H32C	109.5	C66B—C61B—C62B	105.9 (14)
H32A—C32—H32C	109.5	C66B—C61B—P2	114.9 (12)
H32B—C32—H32C	109.5	C62B—C61B—P2	122.1 (11)
C4—C41—H41A	109.5	C66B—C61B—H61B	104
C4—C41—H41B	109.5	C62B—C61B—H61B	104
H41A—C41—H41B	109.5	P2—C61B—H61B	104
C4—C41—H41C	109.5	C63B—C62B—C61B	115.7 (11)
H41A—C41—H41C	109.5	C63B—C62B—H62C	108.3
H41B—C41—H41C	109.5	C61B—C62B—H62C	108.3
C4—C42—H42A	109.5	C63B—C62B—H62D	108.3
C4—C42—H42B	109.5	C61B—C62B—H62D	108.3
H42A—C42—H42B	109.5	H62C—C62B—H62D	107.4
C4—C42—H42C	109.5	C62B—C63B—C64B	114.0 (14)
H42A—C42—H42C	109.5	C62B—C63B—H63C	108.7
H42B—C42—H42C	109.5	C64B—C63B—H63C	108.7
C56A—C51A—C52A	111.3 (2)	C62B—C63B—H63D	108.7
C56A—C51A—P2	112.73 (18)	C64B—C63B—H63D	108.7
C52A—C51A—P2	110.16 (17)	H63C—C63B—H63D	107.6
C56A—C51A—H51A	107.5	C65B—C64B—C63B	111.8 (14)
C52A—C51A—H51A	107.5	C65B—C64B—H64C	109.3
P2—C51A—H51A	107.5	C63B—C64B—H64C	109.3
C53A—C52A—C51A	111.3 (2)	C65B—C64B—H64D	109.3
C53A—C52A—H52A	109.4	C63B—C64B—H64D	109.3
C51A—C52A—H52A	109.4	H64C—C64B—H64D	107.9
C53A—C52A—H52B	109.4	C64B—C65B—C66B	110.9 (14)
C51A—C52A—H52B	109.4	C64B—C65B—H65C	109.5
H52A—C52A—H52B	108	C66B—C65B—H65C	109.5
C54A—C53A—C52A	111.0 (2)	C64B—C65B—H65D	109.5
C54A—C53A—H53A	109.4	C66B—C65B—H65D	109.5
C52A—C53A—H53A	109.4	H65C—C65B—H65D	108.1
C54A—C53A—H53B	109.4	C65B—C66B—C61B	117.4 (12)
C52A—C53A—H53B	109.4	C65B—C66B—H66C	107.9
H53A—C53A—H53B	108	C61B—C66B—H66C	107.9
C53A—C54A—C55A	110.3 (2)	C65B—C66B—H66D	107.9
C53A—C54A—H54A	109.6	C61B—C66B—H66D	107.9
C55A—C54A—H54A	109.6	H66C—C66B—H66D	107.2
C53A—C54A—H54B	109.6	P2—B1—H1A	109.5
C55A—C54A—H54B	109.6	P2—B1—H1B	109.5
H54A—C54A—H54B	108.1	H1A—B1—H1B	109.5
C54A—C55A—C56A	110.9 (2)	P2—B1—H1C	109.5
C54A—C55A—H55A	109.5	H1A—B1—H1C	109.5
C56A—C55A—H55A	109.5	H1B—B1—H1C	109.5
C54A—C55A—H55B	109.5	H7A—O2—H7B	112 (5)
C56A—C55A—H55B	109.5		
			
O1—P1—N4—C3	69.09 (16)	B1—P2—C51A—C52A	56.8 (2)
C21—P1—N4—C3	−166.52 (14)	C56A—C51A—C52A—C53A	−53.8 (3)
C11—P1—N4—C3	−57.50 (15)	P2—C51A—C52A—C53A	−179.52 (18)
O1—P1—N4—C4	−78.11 (16)	C51A—C52A—C53A—C54A	56.1 (3)
C21—P1—N4—C4	46.27 (17)	C52A—C53A—C54A—C55A	−58.2 (3)
C11—P1—N4—C4	155.29 (14)	C53A—C54A—C55A—C56A	58.5 (4)
C4—N4—C3—C32	−65.0 (2)	C54A—C55A—C56A—C51A	−56.4 (3)
P1—N4—C3—C32	145.22 (15)	C52A—C51A—C56A—C55A	53.9 (3)
C4—N4—C3—C31	60.4 (2)	P2—C51A—C56A—C55A	178.20 (19)
P1—N4—C3—C31	−89.35 (19)	C61A—P2—C51B—C52B	−162.1 (11)
C3—N4—C4—C42	122.81 (19)	C61B—P2—C51B—C52B	−159.1 (14)
P1—N4—C4—C42	−89.5 (2)	C51A—P2—C51B—C52B	−13.3 (17)
C3—N4—C4—C41	−108.7 (2)	C12—P2—C51B—C52B	−43.9 (13)
P1—N4—C4—C41	39.0 (2)	B1—P2—C51B—C52B	83.4 (12)
O1—P1—C11—C16	−165.89 (15)	C61A—P2—C51B—C56B	80.7 (12)
N4—P1—C11—C16	−36.79 (17)	C61B—P2—C51B—C56B	83.8 (15)
C21—P1—C11—C16	76.12 (17)	C51A—P2—C51B—C56B	−130 (3)
O1—P1—C11—C12	21.5 (2)	C12—P2—C51B—C56B	−161.1 (10)
N4—P1—C11—C12	150.64 (16)	B1—P2—C51B—C56B	−33.8 (13)
C21—P1—C11—C12	−96.45 (18)	C56B—C51B—C52B—C53B	−60.3 (16)
C16—C11—C12—C13	−4.0 (3)	P2—C51B—C52B—C53B	−178.0 (11)
P1—C11—C12—C13	168.39 (15)	C51B—C52B—C53B—C54B	52 (2)
C16—C11—C12—P2	168.39 (16)	C52B—C53B—C54B—C55B	−46 (2)
P1—C11—C12—P2	−19.2 (3)	C53B—C54B—C55B—C56B	51 (2)
C61A—P2—C12—C13	−123.2 (2)	C54B—C55B—C56B—C51B	−58.9 (19)
C51B—P2—C12—C13	126.5 (7)	C52B—C51B—C56B—C55B	64.2 (15)
C61B—P2—C12—C13	−117.5 (9)	P2—C51B—C56B—C55B	179.0 (11)
C51A—P2—C12—C13	118.32 (17)	C51B—P2—C61A—C66A	160.1 (6)
B1—P2—C12—C13	−1.66 (18)	C61B—P2—C61A—C66A	−1 (7)
C61A—P2—C12—C11	64.1 (2)	C51A—P2—C61A—C66A	151.5 (3)
C51B—P2—C12—C11	−46.2 (7)	C12—P2—C61A—C66A	38.0 (3)
C61B—P2—C12—C11	69.9 (9)	B1—P2—C61A—C66A	−83.7 (3)
C51A—P2—C12—C11	−54.3 (2)	C51B—P2—C61A—C62A	−78.0 (6)
B1—P2—C12—C11	−174.31 (18)	C61B—P2—C61A—C62A	121 (7)
C11—C12—C13—C14	1.7 (3)	C51A—P2—C61A—C62A	−86.7 (3)
P2—C12—C13—C14	−171.94 (17)	C12—P2—C61A—C62A	159.9 (2)
C12—C13—C14—C15	2.0 (3)	B1—P2—C61A—C62A	38.2 (3)
C13—C14—C15—C16	−3.2 (3)	C66A—C61A—C62A—C63A	−59.3 (3)
C14—C15—C16—C11	0.8 (3)	P2—C61A—C62A—C63A	178.9 (2)
C12—C11—C16—C15	2.9 (3)	C61A—C62A—C63A—C64A	58.8 (3)
P1—C11—C16—C15	−170.28 (17)	C62A—C63A—C64A—C65A	−56.2 (4)
O1—P1—C21—C22	−162.56 (17)	C63A—C64A—C65A—C66A	54.3 (4)
N4—P1—C21—C22	68.41 (19)	C62A—C61A—C66A—C65A	57.7 (4)
C11—P1—C21—C22	−41.9 (2)	P2—C61A—C66A—C65A	−179.9 (2)
O1—P1—C21—C26	12.57 (19)	C64A—C65A—C66A—C61A	−55.8 (4)
N4—P1—C21—C26	−116.46 (17)	C61A—P2—C61B—C66B	−160 (8)
C11—P1—C21—C26	133.27 (17)	C51B—P2—C61B—C66B	−179.7 (17)
C26—C21—C22—C23	1.1 (3)	C51A—P2—C61B—C66B	170.2 (14)
P1—C21—C22—C23	176.13 (17)	C12—P2—C61B—C66B	57 (2)
C21—C22—C23—C24	−1.4 (3)	B1—P2—C61B—C66B	−59.9 (18)
C22—C23—C24—C25	1.6 (4)	C61A—P2—C61B—C62B	−30 (5)
C23—C24—C25—C26	−1.5 (4)	C51B—P2—C61B—C62B	−49 (2)
C24—C25—C26—C21	1.3 (4)	C51A—P2—C61B—C62B	−59 (2)
C22—C21—C26—C25	−1.0 (3)	C12—P2—C61B—C62B	−172.6 (18)
P1—C21—C26—C25	−176.41 (18)	B1—P2—C61B—C62B	70 (2)
C61A—P2—C51A—C56A	55.0 (3)	C66B—C61B—C62B—C63B	−48 (2)
C51B—P2—C51A—C56A	22 (2)	P2—C61B—C62B—C63B	178.0 (16)
C61B—P2—C51A—C56A	59.8 (9)	C61B—C62B—C63B—C64B	51 (2)
C12—P2—C51A—C56A	173.8 (2)	C62B—C63B—C64B—C65B	−51 (2)
B1—P2—C51A—C56A	−68.1 (2)	C63B—C64B—C65B—C66B	51 (2)
C61A—P2—C51A—C52A	179.9 (2)	C64B—C65B—C66B—C61B	−56 (2)
C51B—P2—C51A—C52A	147 (2)	C62B—C61B—C66B—C65B	52 (2)
C61B—P2—C51A—C52A	−175.3 (9)	P2—C61B—C66B—C65B	−170.6 (14)
C12—P2—C51A—C52A	−61.2 (2)		

### Hydrogen-bond geometry (Å, º)

**Table:**

*D*—H···*A*	*D*—H	H···*A*	*D*···*A*	*D*—H···*A*
O2—H7*B*···O1^i^	0.88 (7)	1.85 (7)	2.722 (4)	167 (6)
O2—H7*A*···O1	0.85 (5)	1.95 (5)	2.768 (4)	163 (5)
C51*A*—H51*A*···O1	1.00	2.28	3.083 (3)	136
C61*A*—H61*A*···O1	1.00	2.31	3.057 (5)	130

Symmetry code: (i) −*x*+1, −*y*+1, −*z*+1.
